# Screening and Grading of Textural Interface Opacities in DSAEK Grafts with the M-TIO Scale for Predicting Visual Outcomes

**DOI:** 10.3390/diagnostics15101241

**Published:** 2025-05-14

**Authors:** Marina S. Chatzea, George D. Kymionis, Dionysios G. Vakalopoulos, Robert C. O’Brien, Daniella Mora, Katrina Llanes, Elizabeth Fout, William Buras, Concetta Triglia, Rahul S. Tonk, Sonia H. Yoo

**Affiliations:** 1 1st Department of Ophthalmology, “G. Gennimatas” Hospital, National and Kapodistrian University of Athens, 11527 Athens, Greece; marinachatzea@gmail.com (M.S.C.); dionisis.vakalopoulos@gmail.com (D.G.V.); 2 Miller School of Medicine, University of Miami, Miami, FL 33136, USA; robrien@med.miami.edu; 3 Beauty of Sight, Bascom Palmer Eye Bank, Miami, FL 33136, USA; da651115@ucf.edu (D.M.); knl39@med.miami.edu (K.L.); efcaraza@med.miami.edu (E.F.); wbb25@med.miami.edu (W.B.); ctriglia@med.miami.edu (C.T.); 4 Corneal and External Diseases, Bascom Palmer Eye Institute, School of Medicine, University of Miami Miller, Miami, FL 33136, USA; rtonk@med.miami.edu (R.S.T.); syoo@med.miami.edu (S.H.Y.)

**Keywords:** DSAEK, interface, opacities, wave-like deposits, OCT, TIO, M-TIO

## Abstract

**Background**: Textural Interface Opacities (TIOs) following Descemet’s Stripping Automated Endothelial Keratoplasty (DSAEK) have become a notable postoperative concern. Several studies have attempted to identify associations between TIO development and intraoperative factors, including fluid dynamics, irregular stromal surfaces, viscoelastic usage, and recipient immunological responses. Despite these efforts, the precise etiology of TIO remains uncertain. TIO has not been considered predictable in the preoperative setting. Its detection has relied exclusively on slit-lamp biomicroscopy, a subjective approach lacking standardized diagnostic criteria, which limits diagnostic reliability and is highly susceptible to interobserver variability. **Methods**: Optical Coherence Tomography (OCT) images of DSAEK-processed corneal grafts, prepared using the same microkeratome and technique for transplantation at the Bascom Palmer Eye Institute, underwent blinded analysis using a newly developed grading scale termed “M-TIO”. This analysis focused on DSAEK-processed grafts OCT images to evaluate and categorize the occurrence of TIO and assess the final visual acuity of the patients at the 1-year postoperative evaluation. **Results**: Based on the results, the M-TIO grading scale demonstrated strong predictive value, with higher grades on OCT of DSAEK lenticules consistently associated with worse postoperative visual acuity. The study included 221 donor corneas transplanted from 2019 to 2023. Greater TIO based on the “M-TIO” grading scale was associated with worse recipient logMAR VA (Mean 0.151 [99% CI: 0.077 to 0.225] for corneas with no TIO, increased to 0.680 [99% CI: 0.532 to 0.828] for corneas with the greatest TIO grade). These findings highlight the clinical utility of the M-TIO scale as an objective and reliable preoperative tool for assessing graft quality and predicting postoperative visual outcomes. **Conclusions**: This study introduces the “M-TIO” grading scale, which provides a standardized and objective method for evaluating Textural Interface Opacities in DSAEK grafts prior to transplantation. Our results demonstrate a clear association between the severity of TIO as graded by the M-TIO scale, and postoperative visual outcomes, with higher TIO grades correlating with worse visual acuity, emphasizing its value in improving graft selection, and clinical decision-making in DSAEK.

## 1. Introduction

**Figure 1:**
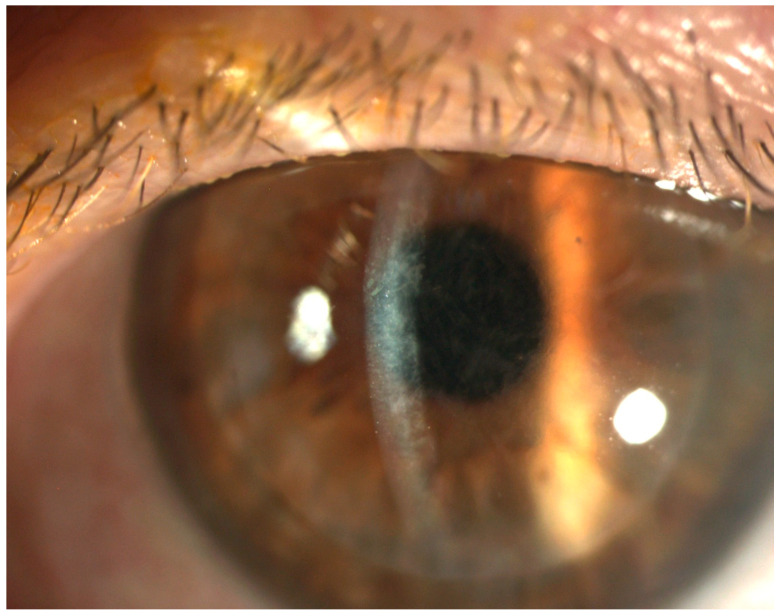
Slit-lamp photograph following DSAEK depicting the diffuse Textural Interface Opacities.

Endothelial Keratoplasty (EK) has become the preferred surgical technique for managing corneal endothelial dysfunction. In 2023, Descemet Membrane Endothelial Keratoplasty (DMEK) emerged as the most frequently performed keratoplasty procedure in the United States, with a total of 17,116 cases. However, it was closely followed by Descemet Stripping Automated Endothelial Keratoplasty (DSAEK), which accounted for 16,207 cases, according to the Eye Bank Association of America’s statistical report, highlighting its continued clinical significance [1,2]. It has been reported that ultrathin Descemet Stripping Automated Endothelial Keratoplasty (UT-DSAEK) offers significantly better visual outcomes compared to standard DSAEK and approaches the visual acuity results of Descemet Membrane Endothelial Keratoplasty (DMEK) while maintaining the similarly low postoperative complication rates associated with conventional DSAEK surgery [3,4,5,6,7]. Compared to traditional penetrating keratoplasty, DSAEK offers several advantages, including faster visual recovery, improved refractive stability and outcomes, better wound integrity, and a lower risk of complications like suprachoroidal hemorrhage [8]. Despite these benefits, DSAEK is associated with certain complications, such as graft dislocation, rejection, pupillary block, epithelial downgrowth, and interface irregularities [8,9,10]. Terms like “Textural Interface Opacities”, “interface wave-like deposits”, “reticular haze”, and “ground glass interface haze” are often used interchangeably in the literature to describe the abnormal accumulation of inert material at the graft–host interface, which can result in significant visual disturbances (Figure 1) [11,12,13,14]. These opacities are characterized by the accumulation of inert material at the graft–host interface, which can manifest as diffuse, central, often undulating grayish opacities observable via slit-lamp examination. Although the exact nature of this material remains uncertain, potential causes include retained viscoelastic and irregular stromal surfaces [13,14,15]. Textural Interface Opacities (TIOs), though relatively rare, are a notable complication that can impair visual acuity and, in persistent cases, require additional surgical intervention [16]. However, long-term follow-up studies suggest that TIO tends to diminish over time, often resulting in gradual improvement in visual acuity and reducing the need for additional surgical intervention [10,17].

Optical Coherence Tomography (OCT) is an essential instrument for evaluating DSAEK corneal grafts preoperatively and assessing the graft–host junction postoperatively [13,18,19]. Preoperatively, OCT enables precise measurement of donor graft thickness, which is crucial for predicting postoperative visual outcomes. Studies have shown that thinner grafts are associated with better visual acuity, highlighting the importance of accurate thickness evaluation [3,4,5,6]. Postoperatively, OCT facilitates the assessment of graft adherence and identification of complications such as graft detachment or interface opacities [19,20]. The PIONEER study utilized intraoperative Optical Coherence Tomography (iOCT) to explore the relationship between central transient interface fluid and the subsequent development of postoperative TIO [14]. Interestingly, TIO does not always occur in procedures where viscoelastic agents are used; conversely, TIO can also appear in cases where no viscoelastic is employed. Although irregular stromal cuts resulting in surface fibers have already been associated with TIO [15], the evaluation of the DSAEK graft interface has been primarily performed using slit-lamp examination. The current standard of care relies on visual inspection to detect interface opacities following the microkeratome cut. However, this method has limitations, as opacities may not always be readily visible, particularly for inexperienced evaluators, and the assessment remains subjective and lacks standardization, leading to potential variability in interpretation. In this study, we developed the “M-TIO” grading scale to assess DSAEK-processed corneal grafts preoperatively with OCT imaging, aiming to establish a screening method for TIO to predict visual outcomes in transplanted patients.

## 2. Materials and Methods

### 2.1. DSAEK Graft Preparation

This single-center retrospective study was conducted over a five-year period, spanning from January 2019 to December 2023. It involved a detailed review and analysis of Optical Coherence Tomography (OCT) images obtained from Descemet Stripping Automated Endothelial Keratoplasty (DSAEK)-processed corneal grafts. All donor tissues were prepared at the Beauty of Sight, Florida Lions Eye Bank, in Miami, Florida using a standardized and consistent protocol involving a rotated microkeratome system (Moria ALTK CBm Combo system; Moria SA, Antony, France). Donor corneal tissues were initially preserved in hypothermic storage at a controlled temperature range of 2–8 °C, in accordance with established guidelines to maintain endothelial cell viability. Before lamellar cutting, each donor cornea was allowed to equilibrate to room temperature to ensure consistent biomechanical properties, thereby improving the accuracy and reliability of the microkeratome dissection. Once the tissue was mounted on the artificial anterior chamber (AAC system, Moria SA, Antony, France), the chamber pressure was adjusted and confirmed to provide the necessary corneal rigidity and surface stability during the cutting process. The central corneal thickness was measured using anterior segment OCT both before and after the microkeratome pass. The appropriate microkeratome head size was chosen based on the donor’s central corneal thickness and the target thickness for the DSAEK graft. A single, slow-pass microkeratome dissection technique was used for all graft preparations to improve consistency and reduce variability in graft thickness [21,22,23].

### 2.2. Data Analysis

**Figure 2:**
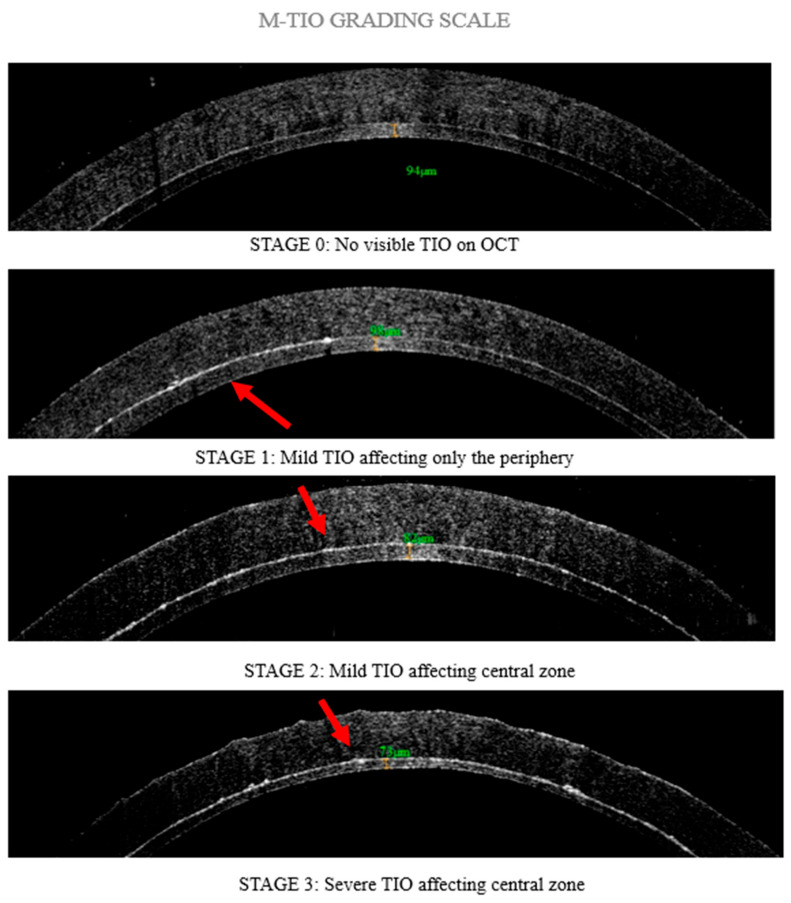
“M-TIO” Grading Scale of DSAEK grafts assessing textural interface opacities (TIO) prior to transplantation. Red arrows indicate irregular hyperreflective areas representing TIO in the graft interface.

Based on prior research indicating that TIO manifests as hyperreflectivity on the donor surface in OCT images [11,14], a four-stage grading scale termed “M-TIO” was established to classify TIO based on the extent of hyperreflectivity. In Stage 0, there is no visible TIO on OCT. Stage 1 involves mild TIO that affects only the periphery of the cornea, specifically beyond the central 4 mm zone. In Stage 2, mild TIO affects the central zone of the cornea, defined as the 4 mm area that represents the average mesopic pupil of adults older than 65 years old [24]. Finally, Stage 3 is characterized by severe TIO affecting the central zone, presenting as a thicker hyperreflective area (Figure 2). All OCT images of DSAEK-processed corneal grafts were evaluated in a blinded manner by three independent corneal specialists to assess the significance of TIO in preoperative evaluations. Following this assessment, the visual acuity outcomes of patients who received the corresponding DSAEK grafts were recorded at the 1-year postoperative evaluation. All procedures were performed using a standardized surgical technique by expert corneal specialists in accordance with the standard of care at Bascom Palmer Eye Institute. Data collection included clinical profiles of the patients, imaging findings, management strategies, and final visual and clinical outcomes. Recipients who received ultra-thin DSAEK grafts <100 μm, for the treatment of Fuchs corneal dystrophy were included in the study, while patients with ocular comorbidities, complicated DSAEK procedures, and DSAEK grafts >100 μm [3,4,5,6], which could negatively impact visual outcomes, were excluded.

In this study, we selected to evaluate pinhole visual acuity (PHVA) instead of best-corrected visual acuity (BCVA) as our primary outcome measure. This decision was based on the recognition that postoperative irregular astigmatism, which may result from factors including interface irregularities or healing responses, cannot always be fully corrected with spectacles or standard refractive methods. Additionally, visual disturbances like glare and halos [25,26], which could be associated with TIO, may persist despite optimal refractive correction and are not assessed by BCVA. PHVA effectively reduces the impact of refractive errors and higher-order aberrations, providing a more accurate representation of the potential visual acuity in the presence of such irregularities [27,28,29]. This approach aligns with clinical practices where pinhole testing is employed to differentiate between refractive and non-refractive causes of visual impairment, particularly in conditions involving corneal irregularities.

### 2.3. Statistical Analysis

Statistical analyses were performed using R version 4.4.2 with the emmeans, eye, lme4, lmtest, performance, and tidyverse packages. More specifically, the emmeans package was used to estimate marginal means and perform pairwise comparisons for the logMAR PHVA values across TIO grades. The lme4 package facilitated the implementation of linear mixed models (LMMs) to account for random intercepts for donors and recipients. The lmtest package was used for hypothesis testing and model diagnostics. The performance package was employed to evaluate the quality of the fitted models. The tidyverse package was utilized for data manipulation, cleaning, and visualization. The logarithm of the minimum angle of resolution (logMAR) PHVA, as determined by TIO grade, was estimated using a linear mixed model (LMM) with random intercepts for donors and recipients. Tukey’s method was used to adjust for multiple comparisons when comparing estimated mean logMAR PHVA among the TIO grades and R2 was calculated using the method of Nakagawa and Schielzeth [30]. A two-sided *p*-value < 0.01 was considered statistically significant.

## 3. Results

There were 221 eyes included in the analysis after excluding glaucomatous eyes and eyes affected by other comorbidities. Among these 221 eyes, the distribution of Textural Interface Opacity (TIO) grades was as follows: 44 eyes (20%) were classified as grade 0, 110 eyes (50%) as grade 1, 56 eyes (25%) as grade 2, and 11 eyes (5%) as grade 3, as most of the latter were excluded from surgery during the slit-lamp evaluation of the DSAEK processed grafts. Postoperative PHVA at the 1-year follow-up was found to decline progressively with increasing TIO grade. Specifically, the estimated mean logMAR PHVA at one year postoperatively was 0.151 (99% Confidence Interval [CI]: 0.077 to 0.225) for eyes with TIO grade 0, 0.311 (99% CI: 0.264 to 0.359) for grade 1, 0.424 (99% CI: 0.358 to 0.490) for grade 2, and 0.680 (99% CI: 0.532 to 0.828) for grade 3 (Table 1). Since higher logMAR values indicate poorer visual acuity, these results demonstrate a clear trend: eyes with higher TIO grades experienced worse visual outcomes. Furthermore, the differences in PHVA between consecutive TIO grades were all found to be statistically significant, with *p*-values less than or equal to 0.002 for every comparison. The magnitude of these differences in visual acuity ranged from a mean difference of 0.113 (99% CI: 0.016 to 0.210) to 0.529 (99% CI: 0.331 to 0.727), indicating not only statistical significance but also clinical relevance (Table 2). The marginal R^2^ value for the regression model used in this analysis was 0.301, meaning that approximately 30% of the variation in PHVA outcomes can be explained by the assigned TIO grade. This finding supports the conclusion that the severity of TIO, as graded by the proposed system, is an important and quantifiable predictor of postoperative visual performance.

## 4. Discussion

OCT provides high-resolution, cross-sectional images of internal biological microstructures, making it an invaluable tool in evaluating eye bank tissue processing techniques for lamellar keratoplasty and optimizing corneal graft thickness prepared by microkeratome [31]. OCT has been widely used in several clinical and experimental studies to identify and characterize interface opacities in recipients of DSAEK corneal grafts. These interface opacities, often subtle and not easily detected through slit-lamp examination, can significantly impact visual recovery and graft clarity, making OCT a critical tool in postoperative assessment [11,13,18]. The PIONEER study, which utilized intraoperative Optical Coherence Tomography (iOCT), demonstrated a significant correlation between the presence of transient interface fluid (TIF) observed during surgery and the subsequent development of TIO in the early postoperative period. These findings suggest that TIF may play a role in the formation of interface opacities following DSAEK. However, the study also emphasized that this relationship is not consistently predictive. Not all cases with intraoperatively detected TIF progressed to develop TIO, and conversely, some instances of TIO occurred in the absence of any observable TIF during surgery. This indicates that while TIF may be a contributing factor, other intraoperative and postoperative variables influence the final interface quality and visual outcomes following DSAEK [14,32].

In our study, we used preoperative OCT images of DSAEK-processed corneal grafts to investigate the presence of TIO, hypothesizing that TIO may result from a combination of irregular donor stroma and the use of viscoelastic agents [15]. Given the variability of TIO, from severe cases requiring surgical management [16] to mild cases that gradually regressed without affecting vision [17], we hypothesized that the degree of irregular donor stroma could predict TIO severity. Accordingly, we developed the “M-TIO” grading scale based on the location and extent of TIO in preoperative OCT images of DSAEK-processed grafts. Two distinct morphological patterns of TIO have been described in the literature: a punctate form, which is typically associated with retained viscoelastic material and tends to be more visually significant, and an elongated form, more commonly attributed to irregularities on the donor stromal surface [11]. Residual stromal fibers resulting from an uneven donor cut may facilitate the retention of viscoelastic material used during the surgical procedure, contributing to the accumulation of visually significant interface deposits. These observations suggest that the microstructural characteristics of the donor tissue may play a critical role in influencing both the likelihood and the severity of TIO following DSAEK.

Among the 221 corneas included in the study, 70% of grafts were classified as grade 0 (*n* = 44) and grade 1 (*n* = 110) for TIO. Only 30%, displayed grade 2 (*n* = 56) or grade 3 (*n* = 11) TIO, which could potentially affect visual acuity and necessitate surgical intervention, aligning with previous reports of the rare occurrence of this condition [11,12,16,17]. It has been demonstrated that a thickened, centrally located hyperreflective area within the stromal layer of a DSAEK-processed corneal graft, as seen on preoperative Optical Coherence Tomography (OCT), has a greater negative effect on postoperative visual acuity than similar irregularities found in the peripheral areas of the graft. The central 4 mm zone of the cornea is significantly important, as it corresponds to the average mesopic pupil size and aligns directly with the visual axis [24]. Irregularities in this zone can cause light scatter and higher-order aberrations [25,26], reducing contrast sensitivity and image clarity, and leading to lower best-corrected visual acuity (BCVA) post-operatively. In contrast, stromal irregularities in the peripheral graft regions are more common but usually have little to no impact on vision, as they lie outside the central visual axis. The high frequency of these peripheral irregularities may explain the wide variation in published data regarding the clinical relevance of TIO, and why its impact on visual outcomes is sometimes reported as minimal and other times as clinically significant.

The correlation between TIO and postoperative visual acuity underscores the need for precise graft assessment to optimize transplantation outcomes. Standardized evaluation methods are essential for accurately identifying and categorizing graft irregularities. The “M-TIO” scale provides an objective approach to assessing graft quality and reducing the risk of TIO-related complications. Incorporating the “M-TIO” grade into DSAEK graft evaluation forms serves as a practical tool for surgeons enabling a systematic approach to optimizing patient-specific visual potential.

Regarding the limitations of our study, the retrospective nature of the analysis prevented us from assessing other visual disturbances, such as glare and halos [25,26], that could affect overall visual quality [33]. Furthermore, only 11 corneal grafts evaluated with grade 3 TIO transplanted in patients were available during this study period, as most were excluded during the slit-lamp evaluation of the DSAEK grafts, which somewhat limited our analysis of the visual acuity outcomes for the corresponding recipients. Finally, there was a learning curve regarding the adaptation of the “M-TIO” grading scale, particularly in recognizing the subtle differences between stages 2 and 3. However, after evaluating the first 30 DSAEK-processed OCT images, the gradings of the three independent evaluators were 95% aligned. Given the high degree of inter-rater agreement observed during the initial calibration phase, characterized by complete concordance among all three evaluators in 95% of cases, and a minimum agreement between two of the three evaluators in all cases, a formal statistical analysis of inter-rater reliability (e.g., Cohen’s or Fleiss’ kappa) was not conducted, as it was deemed methodologically redundant.

## 5. Conclusions

Our study presents a preoperative screening method to assess TIO based on the extent and the area of the irregular donor surface and classify its severity using “M-TIO” grading scale. This approach aims to predict visual outcomes and align with the needs and expectations of potential recipients.

## Figures and Tables

**Table 1:** Mean PHVA by TIO grade (N = 221 Donor Corneas).

		Linear Mixed Model ^1^	
	N = 221	Mean logMAR (99% CI)	*p*-Value
TIO			<0.001
0	44	0.151 (0.077, 0.225)	
1	110	0.311 (0.264, 0.359)	
2	56	0.424 (0.358, 0.490)	
3	11	0.680 (0.532, 0.828)	

Abbreviations: CI = confidence interval; logMAR = logarithm of the minimum angle of resolution; PHVA = pinhole visual acuity; TIO = textural interface opacity. ^1^ Linear mixed model with PHVA regressed on TIO with random intercepts for donor and recipient.

**Table 2:** Mean differences in PHVA by TIO Grade (N = 221 Donor Corneas).

	Linear Mixed Model ^1^	
	Mean Diff logMAR (99% CI ^2^)	*p*-Value ^2^
TIO		
Grade 1–Grade 0	0.160 (0.054, 0.266)	<0.001
Grade 2–Grade 0	0.273 (0.154, 0.392)	<0.001
Grade 2–Grade 1	0.113 (0.016, 0.210)	0.002
Grade 3–Grade 0	0.529 (0.331, 0.727)	<0.001
Grade 3–Grade 1	0.369 (0.183, 0.556)	<0.001
Grade 3–Grade 2	0.256 (0.062, 0.451)	<0.001

Abbreviations: CI = confidence interval; logMAR = logarithm of the minimum angle of resolution; PHVA = pinhole visual acuity; TIO = Textural Interface Opacity. ^1^ Linear mixed model with PHVA regressed on TIO with random intercepts for donor and recipient. ^2^ Tukey’s method was used to adjust the CIs and *p*-values for multiple comparisons.

## Data Availability

The data presented in this study are available on request from the corresponding author. The data are not publicly available due to privacy or ethical restrictions.
